# Transcriptome Analysis of the Carmine Spider Mite, *Tetranychus cinnabarinus* (Boisduval, 1867) (Acari: Tetranychidae), and Its Response to *β*-Sitosterol

**DOI:** 10.1155/2015/794718

**Published:** 2015-05-11

**Authors:** Chunya Bu, Jinling Li, Xiao-Qin Wang, Guanglu Shi, Bo Peng, Jingyu Han, Pin Gao, Younian Wang

**Affiliations:** ^1^College of Biological Science and Engineering, Beijing University of Agriculture, Beijing 102206, China; ^2^Key Laboratory of Urban Agriculture (North), Ministry of Agriculture, Beijing 102206, China; ^3^Plant Science and Technology College, Beijing University of Agriculture, Beijing 102206, China

## Abstract

*Tetranychus cinnabarinus* (Acari: Tetranychidae) is a worldwide polyphagous agricultural pest that has the title of resistance champion among arthropods. We reported previously the identification of the acaricidal compound *β*-sitosterol from* Mentha piperita* and* Inula japonica*. However, the acaricidal mechanism of *β*-sitosterol is unclear. Due to the limited genetic research carried out, we* de novo* assembled the transcriptome of* T. cinnabarinus* using Illumina sequencing and conducted a differential expression analysis of control and *β*-sitosterol-treated mites. In total, we obtained >5.4 G high-quality bases for each sample with unprecedented sequencing depth and assembled them into 22,941 unigenes. We identified 617 xenobiotic metabolism-related genes involved in detoxification, binding, and transporting of xenobiotics. A highly expanded xenobiotic metabolic system was found in mites.* T. cinnabarinus* detoxification genes—including carboxyl/cholinesterase and ABC transporter class C—were upregulated after *β*-sitosterol treatment. Defense-related proteins, such as Toll-like receptor, legumain, and serine proteases, were also activated. Furthermore, other important genes—such as the chloride channel protein, cytochrome* b*, carboxypeptidase, peritrophic membrane chitin binding protein, and calphostin—may also play important roles in mites' response to *β*-sitosterol. Our results demonstrate that high-throughput-omics tool facilitates identification of xenobiotic metabolism-related genes and illustration of the acaricidal mechanisms of *β*-sitosterol.

## 1. Introduction

The carmine spider mite,* Tetranychus cinnabarinus* (Boisduval, 1867) (Acari: Tetranychidae), is one of the most significant species of the genus* Tetranychus*, which includes over 140 species [1, 2]. It belongs to the class Arachnida, infraclass Acari, order Prostigmata, and family Tetranychidae.* T. cinnabarinus* is an economically important pest that infests greenhouse and field crops, such as tomatoes, peppers, cucumbers, strawberries, maize, soy, apples, grapes, and citrus [1].* T. cinnabarinus* is one of the most polyphagous arthropod herbivores known. It has been documented to feed on more than 1,100 plant species belonging to >140 plant families [1]. This mite has a worldwide distribution and has an extensive host plant range throughout China. These spider mites are the “resistance champion” among arthropods, as they have an extreme record of resistance to almost all acaricides [1, 3, 4]. Unreasonable and intensive use of acaricides has accelerated the development of resistance by mites in China. Rapid development, high fecundity, and haplodiploid sex determination may contribute to the rapid evolution of pesticide resistance in this mite [1].

Considering the seriousness of the mite problem, it is necessary to develop alternative acaricides to overcome the resistance of* T. cinnabarinus*. Botanical pesticides are desirable in pest management, as they are often environmentally benign and induce resistance less frequently. *β*-Sitosterol, one of three common phytosterols, was isolated from a petroleum-ether extract of* Sapium baccatum* (Roxb.) leaves and shows high activity in a brine shrimp bioassay with an LC_50_ of 1.58 mg/mL [5]. In addition, *β*-sitosterol has been identified in* Dunalia spinosa* (Meyen) extracts, where it exhibits strong antimicrobial activity [6]. *β*-Sitosterol is present in a variety of plants and is an antitumor, antimicrobial, chemopreventive, and immunomodulatory compound [7, 8]. *β*-Sitosterol is also the active compound of the mulberry plant that induces biting behavior from* Bombyx mori* (Linnaeus) in a low concentration [9]. However, *β*-sitosterol alone has greatly reduced effects on biting behavior of* Antheraea assamensis* (Helfer) [10]. Antifungal and antibacterial activities of *β*-sitosterol have been detected in a methanol extract of dried-ground aerial parts of* Senecio lyratus* (Bullock) [11]. We previously isolated *β*-sitosterol from* Mentha piperita* (Linnaeus) and reported high acaricidal activity (LC_50_, 0.5 mg/mL) [12]. *β*-Sitosterol may thus be a candidate lead botanical compound. However, the acaricidal mechanism of *β*-sitosterol is unclear.

The availability of genome and transcriptome sequences provides unique information and enables investigation of the mode of action of an acaricidal compound and the roles of the gene families involved in pesticide metabolism in spider mites [13–15]. Characterizing the spider mite gene families associated with *β*-sitosterol metabolism is the first step towards a better understanding of the effects of *β*-sitosterol on spider mites. In this study, we performed a detailed analysis of the expression of *β*-sitosterol metabolism-associated gene families in* T. cinnabarinus*.

## 2. Materials and Methods

### 2.1. Carmine Spider Mite Collection and Handling

A colony of* T. cinnabarinus* (Boisduval, 1867) was originally collected from a field in Qingxu County, Taiyuan Municipality, Shanxi Province, China, in the spring of 2004 and had been free of pesticides for >10 years. This insecticide-susceptible strain of* T. cinnabarinus* was maintained at the Key Laboratory of Urban Agriculture (North), Ministry of Agriculture (Beijing, China), at 26 ± 2°C, 60% relative humidity (RH), and a 16 h light: 8 h dark photoperiod under pesticide-free conditions. The mites were reared on* Vigna unguiculata* (L.) leaves at the sixth true-leaf growth stage in a greenhouse. The mites were tested annually for high sensitivity to organophosphate insecticides, using the glass slide-dipping method according to FAO standards.

### 2.2. *β*-Sitosterol Treatment

Our laboratory determined previously that *β*-sitosterol has high acaricidal activity against* T. cinnabarinus*. The changes in* T. cinnabarinus* gene expression were evaluated after exposure to a sublethal concentration (LC_30_) of *β*-sitosterol according to the procedure described by [16, 17]. The LC_30_ is the concentration required to kill 30% of the members of a tested population after a specified test duration and is commonly used in mite pesticide exposure research [18]. The mites were treated with *β*-sitosterol using the glass slide-dipping method according to the FAO [19]. *β*-sitosterol (Sigma-Aldrich, St. Louis, MO, USA) was diluted in sterile distilled water containing 1% Tween 80 (Sigma-Aldrich) and 0.6% DMSO (Sigma-Aldrich). Sterile distilled water with 1% Tween 80 and 0.6% DMSO was used as the control. Killing of the female adults is critical for controlling spider mites. Thus, female adults were used in our experiment. Female adult mites were affixed onto one end of a 10 × 2 cm glass slide using double-sided adhesive tape. The affixed mites were checked under a stereomicroscope, and inactive mites were removed. The mites were dipped individually into *β*-sitosterol solutions for 5 s. Following removal of the slide, the excess solution was absorbed with filter paper. All treated mites were maintained at 26 ± 2°C, 60% RH, and a 16 h light: 8 h dark photoperiod. The dead mites were removed 24 h after treatment, and the live mites were frozen immediately in liquid nitrogen and stored for later extraction of RNA [19]. This experiment was repeated twice.

### 2.3. RNA Extraction, cDNA Library Construction, and Illumina Sequencing

Total RNAs were extracted using TRIzol reagent (Invitrogen, Beijing, China) according to the manufacturer's instructions. Genomic DNA was removed using RNase-free DNase (Takara, Kyoto, Japan). The quantity and quality of total RNA were determined using a NanoDrop 2000 UV-Vis Spectrophotometer (Thermo Scientific, Wilmington, DE, USA), Ultrasec 2100 pro UV/Visible Spectrophotometer (Amersham Biosciences, Uppsala, Sweden), and gel electrophoresis.

Poly-(A) mRNA was enriched and isolated from the high-quality total RNA samples using Magnetic Oligo(dT) Beads, followed by fragmentation of the mRNA with a RNA fragmentation kit (Ambion, Austin, TX, USA). Reverse transcription into cDNA was performed using random hexamers and reverse transcriptase (Promega, Madison, WI, USA), and then second-strand cDNA was synthesized using DNA polymerase I (Invitrogen) and RNaseH (New England Biolabs, Ipswich, MA, USA). After purification with the QIAquick PCR Purification Kit (Qiagen, Valencia, CA, USA), the cDNA fragments were end-repaired and ligated to a Solexa adaptor after adding a single “A” at the 3′ end. The desired size of adaptor-ligated fragment was selected by agarose gel electrophoresis. Polymerase chain reaction (PCR) was performed to selectively enrich and amplify the selected cDNA fragments to construct cDNA libraries for paired-end sequencing using the Illumina HiSeq 2000 (Biomarker Technologies Co., Ltd., Beijing, China).

### 2.4. *De Novo* Assembly and Clustering Analysis

*De novo* assembly of the mRNA-seq reads was performed using the Trinity method (http://trinityrnaseq.sourceforge.net/) after the raw reads were filtered by discarding the adaptor sequences, low-quality sequences (reads with ambiguous “N” bases), and reads in which >10% of the bases had *Q* < 20. An optimized* k*-mer length of 25 was used to assemble the transcriptome. A clustering analysis was then conducted to construct the unigene database.

### 2.5. Functional Annotation

Open reading frames (ORF) of the unigenes were predicted using the “getorf” program in the EMBOSS software package (http://emboss.sourceforge.net/apps/cvs/emboss/apps/getorf.html), and the longest ORF was selected for each unigene. Unigene functions were annotated by a BLASTX search against the nonredundant protein database (http://www.ncbi.nlm.nih.gov/), the SwissProt database (http://www.uniprot.org/), the TrEMBL database (http://www.uniprot.org/), the clusters of orthologous groups (COG) database, (http://www.ncbi.nlm.nih.gov/COG/), and the Kyoto Encyclopedia of Genes and Genomes (KEGG database, http://www.genome.jp/kegg/), using a BLASTN search against the nonredundant nucleotide database (Nt database, http://www.ncbi.nlm.nih.gov/), and by a Blast2GO search against the Gene Ontology (GO) database, (http://www.geneontology.org/) with an *E*-value ≤ 1*e* ^−5^.

### 2.6. Simple Sequence Repeat (SSR) Analysis

The MISA software (MIcroSAtellite identification tool, http://pgrc.ipk-gatersleben.de/misa/) was used to identify potential SSRs from the unigene database. The parameters were set as follows: a minimum of 10 repeats for mononucleotides, six for dinucleotides, and five for tri-, penta-, and hexanucleotide motifs.

### 2.7. Differential Expression of Unigenes

Differential expression of the unigenes between control mites and the *β*-sitosterol-treated mites was analyzed using the IDEG6 software (http://telethon.bio.unipd.it/bioinfo/IDEG6/). In this study, false discovery rate (FDR) ≤ 0.01, a reads per kilobase of exon model per million mapped reads (PRKM) value ≥2, and the absolute value of log_2_ ratio ≥1 were used as the thresholds of significant differences in gene expression between control and *β*-sitosterol treated mites.

### 2.8. Reverse Transcription Quantitative Real-Time PCR (RT-qPCR)

A subset of highly differentially expressed genes was validated by RT-qPCR. Total RNA from the *β*-sitosterol-treated female adult mites and the control female adult mites was extracted as described above, and total RNA quality was high. The genomic DNA was removed, and cDNA was synthesized using a PrimeScript RT reagent kit with the gDNA Eraser (Takara, Dalian, China). qPCR was performed on an ABI 7300 Real-Time PCR System (ABI) using SYBR Premix Ex Taq II (Tli RNaseH Plus) (Takara, Kyoto, Japan). The qPCR was performed as follows: 95°C for 30 s, followed by 40 cycles of 5 s at 95°C and 31 s at 60°C, and a melting curve analysis was conducted from 60 to 95°C. The qPCR primers are listed in Table S1 in Supplementary Material available online at http://dx.doi.org/10.1155/2015/794718. No-reverse-transcription controls and nontemplate controls were included on each plate. Each of the two primer pairs for the tested genes and endogenous references produced a single peak in melting curve analyses and gel electrophoresis and had PCR efficiency close to 100%, as determined using a cDNA dilution series obtained from a single sample.

Transcript levels of the tested genes were determined using the *β-actin* and* a-tub* genes as endogenous references for normalization by the 2^−ΔΔCt^ method. Experiments were repeated three times. Differences in mRNA levels between the *β*-sitosterol-treated and control mites were statistically analyzed using equal or unequal variance *t*-tests, depending on the results of *F*-tests for homoscedasticity.

## 3. Results

### 3.1. Sequence Analysis and* De Novo* Assembly

The transcriptome of *β*-sitosterol-treated female adult and control mites were sequenced using the Illumina HiSeq 2000. We obtained >5.4 G high-quality bases with 98.88% *Q*_20_ bases for each* T. cinnabarinus* sample after removing the dirty reads from the raw reads, an unprecedented sequencing depth (Table 1). The* T. cinnabarinus* contigs had an average GC content of 38.98%.

These high-quality reads were* de novo* assembled using the Trinity method, and 22,941 unigenes with a mean length of 1,468 bp were obtained (Table 2). All unigenes were >200 bp with ≥2000 bp sequences representing the highest proportion, followed by 1000–2000 bp sequences. A total of 11,114 (48.45%) unigenes were longer than 1000 bp, and 5,960 (25.98%) were longer than 2000 bp. In total, 14,831 (64.65%) unigenes were longer than 500 bp. These results show that Illumina sequencing enables rapid capture of a large portion of a transcriptome.

### 3.2. Functional Annotation

All unique unigenes were annotated by a BLAST search against the sequences in the nonredundant database, SwissProt database, TrEMBL database, GO database, COG database, KEGG database, and Nt database with a cut-off* E*-value of 1 × 10^−5^. A total of 12,049 of all (52.52%) unigenes were annotated in our database (Table 3). A wide array of genes was homologous to genes in different classes and different organisms. A total of 11,453 unigenes had matches in the Nr database and 4,754 had matches in the Nt database, whereas 9,259 unigenes yielded a significant hit in the SwissProt database.

**Figure 1:**
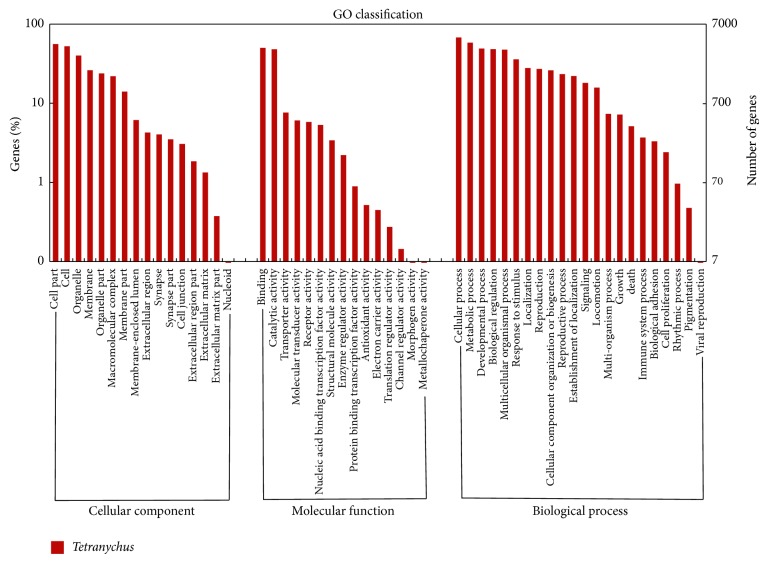
Functional annotation of assembled sequences based on the Gene Ontology (GO) categorization. The *x*-axis indicates the GO categories, which are grouped into three main ontologies: cellular components, molecular functions, and biological processes. The left *y*-axis indicates the percentage of genes in the category, whereas the right *y*-axis indicates the number of genes in each category. “*Tetranychus*” indicates that the unigenes were assembled using the reads from control and *β*-sitosterol-exposed* Tetranychus cinnabarinus*.

The unigenes for the GO analysis were divided into three categories and 53 subcategories: 29.18% unigenes for cellular components (16 subcategories), 14.76% for molecular functions (15 subcategories), and 56.05% for biological processes (22 subcategories) (Figure 1). Some of these unigenes participated in multiple GO terms. The three major subcategories were cellular and metabolic processes in the biological processes category and cell parts in the cellular component category. The smallest subcategories were metallochaperone activity (molecular functions), viral reproduction (biological processes), morphogen activity (molecular functions), and nucleoid (cellular components).

**Figure 2:**
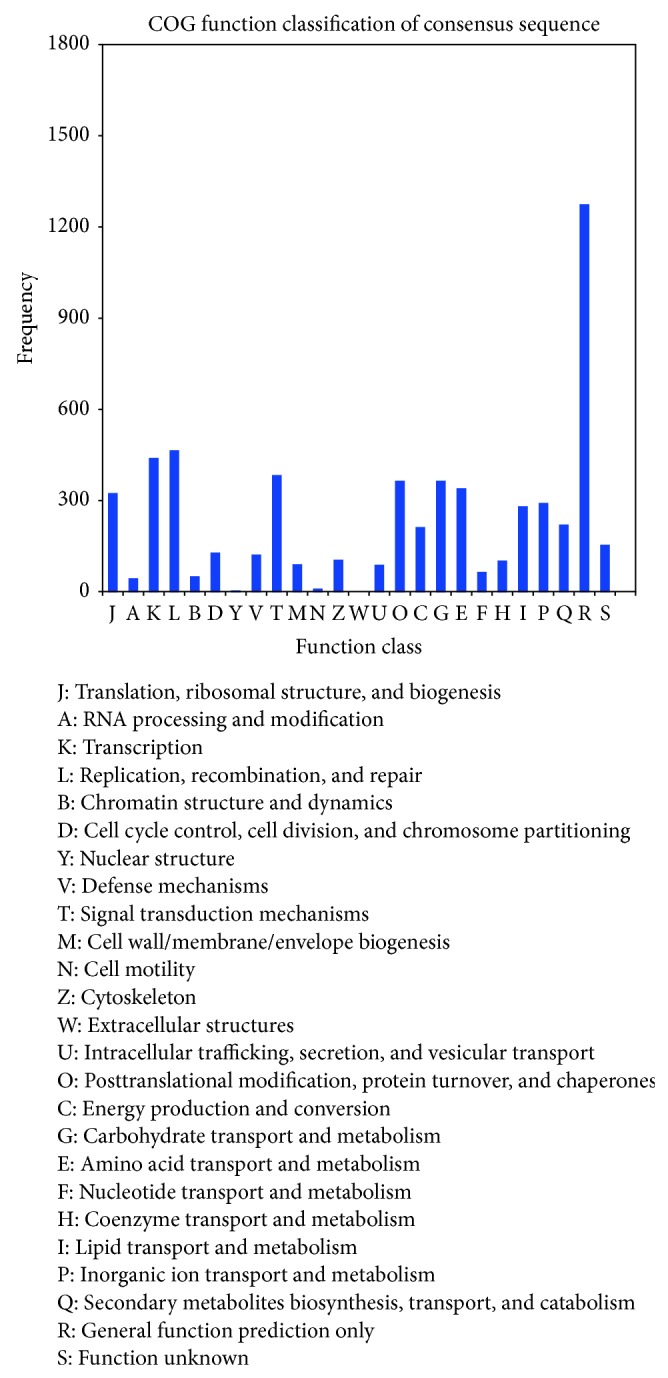
Clusters of orthologous (COG) classification. A total of 4,283 unigenes were grouped into 25 COG classifications. The *y*-axis indicates the number of genes in a specific function cluster. The legend shows the 25 function categories.

In addition, 4,283 unigenes had significant matches in the COG database. Among the 25 COG categories, the “general function prediction” cluster occupied the highest proportion (1273, 21.66%), followed by “replication, recombination, and repair” (463, 7.88%) and “transcription” (438, 7.45%) (Figure 2). The three smallest groups were “extracellular structures,” “cell cycle control,” “cell division and chromosome partitioning,” and “cell motility.”

### 3.3. SSR Discovery

SSRs are widely used for evolution and genetics studies [20, 21]. The spider mite had a relatively low density of microsatellites (Table 4). Even dinucleotide repeats (696, 30.17%), which are typically the most abundant microsatellite type were markedly less frequent than trinucleotides (1583, 68.62%). Tetranucleotide, pentanucleotide, and compound SSRs comprised <1% of all SSRs.

### 3.4. Analysis of Genes Involved in Insecticide Metabolism

To understand how* T. cinnabarinus* metabolizes xenobiotics, such as insecticides and secondary plant chemicals, we identified known genes implicated in digestion, detoxification, and xenobiotic transport (Table 5). The xenobiotic metabolic system of the spider mite was expanded. Eighty-one cytochrome P450 (CYP) genes were detected in the* T. cinnabarinus* transcriptome (Tables S2 and S3). Thirty-five glutathione S-transferases (GSTs) and 28 carboxyl/cholinesterases (CCEs) were identified (Tables S4–S6). In addition, a group of 11 Mu-class GSTs were detected within the family of 35 GSTs. There were also a large number of genes involved in general pesticide detoxification: six catalases, 12 superoxide dismutases, and 28 NADH dehydrogenases.

In addition to metabolizing enzymes, a large number of transporters were also identified. We identified 49 ATP-binding cassette transporters (class C). The MFS family is one of the largest membrane transporter families along with ABC transporters. Seventeen major facilitator superfamily genes were annotated in* T. cinnabarinus*. The transporters were expanded in* T. cinnabarinus*.

The main insecticide targets for commonly used insecticides were identified in the* T. cinnabarinus* transcriptome (Table 5). Nine gamma-aminobutyric acid (GABA) receptors, 15 glutamate-gated chloride channels (GluCls), and 25 nicotinic acetylcholine receptors (nAChR) were identified. One acetylcholinesterase (AChE), 10 voltage-gated sodium channels, and nine cytochromes* b* were also identified. In addition, 14 GABA-gated chloride channels and two ryanodine receptors were identified. These target sequences will facilitate understanding of the development of resistance in these mites and assist in development of new pesticides.

### 3.5. Differential Expression Analysis

**Figure 3:**
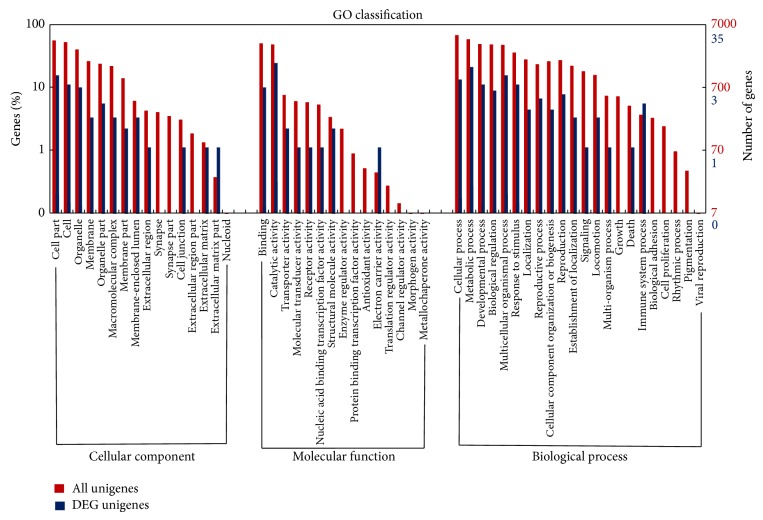
Gene Ontology (GO) functional classification of all and differentially expressed unigenes. “All unigenes” indicates unigenes that were assembled using the reads from control and *β*-sitosterol-exposed mites. “DEG unigene” indicates the unigenes differentially expressed between control and *β*-sitosterol-exposed mites.

**Figure 4:**
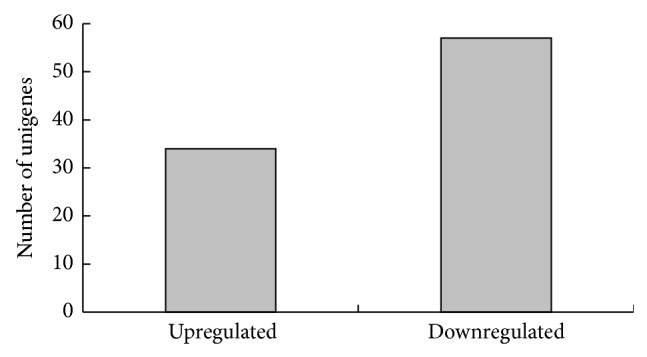
Gene expression profiles of control and *β*-sitosterol-exposed mites.

The differences in gene expression levels between control and *β*-sitosterol-treated mites after *β*-sitosterol exposure were analyzed using the IDEG6 software. FDR ≤ 0.01, PRKM value ≥ 2, and the absolute value of the log_2_ ratio ≥ 1 were used as the threshold to determine significant differences in gene expression. Over half of all differentially expressed genes were assigned to a GO category (Figure 3). We found 34 genes upregulated and 57 downregulated in the *β*-sitosterol-treated mites compared to in the control mites (Figure 4, Table S7). The highly differentially expressed genes are listed in Table 6. Nine of the differentially expressed genes were annotated using the KEGG database, and eight pathways were involved. Pathways, such as glycan degradation, fatty acid biosynthesis, antigen processing and presentation, extracellular matrix- (ECM-) receptor interactions, and oxidative phosphorylation, play roles in detoxifying *β*-sitosterol.

The expression profiles of some insecticide metabolic genes changed, such as CCE, ABC transporter class C, carboxypeptidase, fatty acid synthase, cytochrome* b*, Toll-like receptor, chloride channel proteins, and serine proteases (Table 6). Among these differentially expressed genes, detoxification enzymes, transporters, small secreted proteins, membrane-binding proteins, and chitin proteins featured prominently.

### 3.6. RT-qPCR Validation Study

A subset of 40 highly differentially expressed genes between the *β*-sitosterol-treated and control mites identified by transcriptomic analyses were validated by RT-qPCR (Table 6). Thirty-six of the forty tested genes showed agreement with the transcriptomic results. The expression profiles of these 40 genes are shown in Table 6, including CCE, ABC transporter class C, peritrophic membrane chitin binding protein, calphostin, fatty acid synthase, chloride channel protein, cytochrome* b*, zinc carboxypeptidase, putative transcription factor, and Toll-like receptor.

## 4. Discussion

### 4.1. Exploring the Spider Mite Transcriptome

The* Tetranychus urticae* (Koch, 1836) genome (strain London) has been sequenced, and the size of the genome was ~90 Mb. Thus, the* T. urticae* genome is the smallest arthropod genome sequenced to date [1]. However, the* T. cinnabarinus* genome remains unknown. According to available* T. cinnabarinus* gene sequence information, there are interesting differences between* T. cinnabarinus* and* T. urticae*. Here, we performed* de novo* assembly of the* T. cinnabarinus* transcriptome using Illumina paired-end sequencing technology. In total, we obtained >5.4 G high-quality bases for each* T. cinnabarinus* sample with unprecedented sequencing depth (Table 1). These high-quality reads were* de novo* assembled, and 22,941 unigenes were obtained with a mean length of 1,468 bp (Table 2). The insecticide resistance-related genes of* T. cinnabarinus* were recently analyzed [22]. Here, we focused on the differential expression of* T. cinnabarinus* genes induced by *β*-sitosterol treatment. We explored genes involved in xenobiotic metabolism and *β*-sitosterol detoxification in particular.

The* T. cinnabarinus* sequences we obtained were similar to those of other arthropods, some species of insects, and other organisms [17, 23, 24]. The most related organisms are* T. urticae* and* Panonychus citri* (McGregor, 1916) [25, 26], particularly* T. urticae*. More than 70% of the unigenes in our dataset significantly matched the* T. urticae* genome database following alignment of* T. cinnabarinus* transcriptome to the* T. urticae* genome. As mites have some of the smallest genomes in the animal kingdom [23],* T. cinnabarinus* has a relatively small microsatellite density, which is correlated with its compact size [1, 22]. A significant portion of the unigenes had novel sequences with unknown function. These results show that our knowledge of mite genes is limited, and further research is needed to characterize mite genes and explore their functions [23].

### 4.2. Spider Mite Metabolism of Xenobiotics

*T. cinnabarinus* has an extensive host plant range and an extraordinary ability to develop resistance to pesticides. A link between the broad response to diverse synthetic and natural xenobiotics and the evolution of pesticide resistance has been proposed [13, 27, 28]. However, the specific metabolic processes involved in digesting, detoxifying, and transporting xenobiotics have been unclear to date. Increasing the metabolic capability of detoxifying systems and reducing xenobiotic target site sensitivity are thought to be major mechanisms of development of xenobiotic resistance [27]. Amplification, overexpression, and variations in the coding sequences of the three major metabolic enzymes—CYP, CCEs, and GSTs—have been reported [27]. This spider mite has a unique xenobiotic metabolic system and a considerably greater number of major metabolic enzymes compared to insects [1, 29]. This was also evident in our transcriptome data. Eighty-one CYPs, twenty-eight CCEs, and thirty-five GSTs were identified in the* T. cinnabarinus* transcriptome. Six major GST subclasses occur in insects, the sigma, omega, theta, and zeta, and the insect-specific delta and epsilon subclasses [30, 31]. The delta and epsilon GST classes seem to be involved in detoxifying xenobiotics. However, a group of Mu-class GSTs, but not epsilon-class GSTs, was found in* T. cinnabarinus*. Mu-class GSTs are believed to be vertebrate-specific [1] and may perform the role of epsilon-class GSTs in* Tetranychus* taxa [32]. CYP genes are assigned to four clades, the CYP2, CYP3, and CYP4 clades and the mitochondrial CYP clade (CYP M) [33]. Expansion of clade 2 and contraction of clade 3 were observed in* Tetranychus* taxa compared to that in insects. The CYP3 and CYP4 clades in most insect species are involved in detoxifying xenobiotics and phytotoxins [34], whereas these functions might be fulfilled by the CYP2 and CYP4 clades in* Tetranychus* taxa [22].

The insect metabolic detoxification system typically includes phase I and phase II metabolizing enzymes as well as phase III transporters, which are induced after xenobiotic exposure. Expansion of the transporters was also found in* T. cinnabarinus*. Thirty-nine multidrug resistance proteins belonged to the ATP-binding cassette transporters (class C) [1]. In this study, 49 ATP-binding cassette transporters (class C) were found in* T. cinnabarinus*, which far exceeds the number (9–14) found in crustaceans, insects, vertebrates, and nematodes [1]. In brief, many gene families from transcription factors to effector genes involved in detoxification, binding, and transport of xenobiotics were characterized in this study. The expansion of the xenobiotic metabolic system suggests a mechanism for the high level of resistance of spider mites to almost all acaricides.

The main targets of commonly used insecticides were identified in* T. cinnabarinus* (Table 5). GABA receptors, GluCls, and nAChR are important ligand-gated ion channels [35]. The GABA receptor is the main target of phenylpyrazole insecticides, such as fipronil, abamectin, and cyclopentyl diene insecticides [22, 36]. We identified nine GABA receptors. GluCls are the ideal targets for highly selective insecticides, as they are found only in invertebrates [22]. Insecticides targeting GluCls include abamectin, ivermectin, fipronil, and the indole diterpenoid compound nodulisporic acid [22]. Fifteen GluCls were identified in* T. cinnabarinus* in this study. nAChR is another important selective insecticide target. Newly developed insecticides, including nereistoxin, neonicotinoid, and the biological insecticide spinosad, specifically target the insect nAChR [35]. AChE is the major target for organophosphates and carbamates. Insecticides targeting AChE account for more than one-third of insecticide sales worldwide [37]. AChE mutations and their combinations can result in varying levels of resistance to many organophosphate and carbamate insecticides [38]. In this study, a single AChE gene was identified in* T. cinnabarinus*. Voltage-gated sodium channels are frequently studied voltage-gated ion channels and are targets of pyrethroids and fenpropathrin [39]. Here, 10 sodium channel genes were identified. Full-length target gene sequences can be amplified based on the partial sequence obtained from the transcriptome. Abundant information regarding the sequences of these pesticide targets will improve our understanding of the molecular mechanisms of xenobiotic resistance. It may be possible to design new effective pesticides in the near future.

### 4.3. Analysis of Differentially Expressed Genes Related to *β*-Sitosterol Metabolism

*β*-Sitosterol is present in a variety of plants and has antitumor, antimicrobial, chemopreventive, and immunomodulatory activities [7, 8]. We reported previously identification of *β*-sitosterol in acaricidal fractions of* Mentha piperita* [12]. *β*-Sitosterol has also been detected in* Inula japonica* Thunberg and had high acaricidal activity against* T. cinnabarinus* [40]. *β*-Sitosterol may be a candidate novel botanical pesticide. However, the acaricidal mechanism of action of *β*-sitosterol is unclear.

Large-scale sequencing technologies, such as Illumina Solexa, enable evaluation of transcriptome dynamics under various conditions without sophisticated normalization of datasets [41]. Investigating fine transcriptome variations after short-term exposure of insects to xenobiotics could provide information useful for identifying novel transcripts involved in the response to xenobiotics and insecticide tolerance [42]. We identified genes of many pathways involved in the *β*-sitosterol-related response, such as glycan degradation, fatty acid biosynthesis, antigen processing and presentation, ECM-receptor interactions, and oxidative phosphorylation. Among these differentially expressed genes, those encoding detoxification enzymes, defense-related factors, silk proteins, and several of unknown function featured prominently.

Exposing living organisms to xenobiotics induces a coordinated transcriptional response that regulates the expression of enzymes and transporters to facilitate detoxification [17, 43, 44]. Some acaricidal components alter the activities of mite detoxification enzymes [22]. In this study, expression of the CCE and transporter ATP-binding cassette class C gene detoxification enzymes was upregulated after exposure of* T. cinnabarinus* to *β*-sitosterol. It was demonstrated that many ATP-binding cassette genes respond transcriptionally to xenobiotic exposure [4]. Moreover, the broad plant host range and high pesticide resistance levels of* T. urticae* are associated with lineage-specific expansion of detoxification genes [4]. Exposing mites to *β*-sitosterol resulted in upregulation of the expression of CCE and transporters that facilitate detoxification.

Defense-related proteins, such as Toll-like receptor, legumain, and serine proteases, responded significantly to the *β*-sitosterol treatment. After pathogen infection, the expression levels of a coordinated set of* Caenorhabditis elegans* (Maupas, 1900) defense genes involving proteolysis, cell death, and the stress response was altered [45, 46]. The recognition of different pathogen classes via germline-encoded proteins, such as the Toll-like receptors, is the first line of defense in vertebrates [47]. Endosome/lysosome-located proteases play key roles in defense, and these proteases may be attractive drug targets. Asparagine endopeptidase (AEP), or legumain, is an unusual lysosomal cysteine protease [48]. This enzyme has been linked to a diverse range of biological phenomena, including plant toxic protein processing, antigen processing and presentation [49], neuronal cell death [50], and tumor invasion [51]. Some endosome/lysosome-located serine proteases have important roles in the defense system [52]. However, further work is needed to determine the roles of Toll-like receptors, legumain, and serine proteases in the response of* T. cinnabarinus* to *β*-sitosterol.

Silk webs protect spider mites against predators, rain, and wind [53]. Silk also plays an important role during collective migration by aerial dispersal or by walking [53]. Seventeen fibroin genes have been identified in the* T. urticae* genome [1]. Our results demonstrate that fibroin gene expression was downregulated during the *β*-sitosterol response. Our previous studies also show that exposure of mites to xenobiotics can decrease silk production. Environmental stressors, such as pesticides, can be detrimental to organisms and reduce their activities [54].

The expression profiles of several potential pesticide target genes, such as cytochrome* b* and the chloride channel protein, were altered. Cytochrome* b* is a target of bifenazate and acequinocyl [55]. Moreover, a proportion of the differentially expressed genes has not been annotated. Future research involving cloning and functionally analyzing of these significantly altered genes could facilitate discovery of additional factors involved in pesticide metabolism.

## 5. Conclusion

We identified 22,941 unigenes by Illumina-sequencing the transcriptome of* T. cinnabarinus*, of which 12,049 were annotated. Abundant xenobiotic metabolism-related genes were identified in* T. cinnabarinus*. In total, 81 P450-related genes, 28 CCEs, 35 GSTs, nine GABA receptors, 15 GluCls, one AChE, 10 voltage-gated sodium channels, nine cytochromes* b*, 25 nAChRs, six catalases, 12 superoxide dismutases, and 28 NADH dehydrogenases were identified from the* T. cinnabarinus* transcriptome. Expansion of the xenobiotic metabolism system was found, which may be responsible for the high level of resistance to almost all acaricides of* T. cinnabarinus*. The* T. cinnabarinus* detoxification system—including CCE and ABC transporter class C—was upregulated after *β*-sitosterol treatment. Defense-related proteins, such as Toll-like receptors, legumain, and serine proteases, were also activated. Furthermore, other important proteins—such as chloride channel protein, cytochrome* b*, carboxypeptidase, peritrophic membrane chitin-binding protein, calphostin, and transcription factors—may also play important roles in the interaction between *β*-sitosterol and spider mites. Further work is needed to determine their roles in the response of* T. cinnabarinus* to *β*-sitosterol treatment.

## Data Deposition

Our sequencing data has been deposited in SRA (SRP043517).

## Supplementary Material

The supplementary materials include seven supplementary tables. In the supplementary tables, the details of primers used for RT-qPCR analyses are provided. Furthermore, the gene numbers of CYP, GSTs and CCEs of Tetranychus cinnabarinus, and comparison of CYP and GSTs among different subgroups are illustrated. In the supplementary tables, gene numbers of differential expressed unigenes of Tetranychus cinnabarinus after exposure to β-sitosterol are also provided.

## Figures and Tables

**Table 1:** Summary of Illumina transcriptome sequencing for *Tetranychus cinnabarinus*.

	Control	*β*-Sitosterol-exposed
Total number of reads	27,495,256	26,870,180
Total number of nucleotides (bp)	5,553,615,807	5,427,335,287
*Q*_20_ percentage	98.88%	98.88%
GC percentage	38.87%	39.10%

**Table 2:** The length distribution of unigenes after *de novo* assembly using the reads from control and *β*-sitosterol-exposed *Tetranychus cinnabarinus*.

Length range	The unigene number	Percentage
200–300	4,495	19.59%
300–500	3,615	15.76%
500–1000	3,717	16.20%
1000–2000	5,154	22.47%
2000+	5,960	25.98%
Total number of unigenes	**22,941**	
Total length of unigenes	33,696,949	
*N*_50_ length of unigenes	2,534	
Mean length of unigenes	1,468.85	

**Table 3:** Function annotation of the *Tetranychus cinnabarinus* transcriptome.

Annotated databases	All sequences	≥300 bp	≥1000 bp
COG	4,283	552	3,626
GO	7,180	1,184	5,702
KEGG	4,455	633	3,676
Swissprot	9,259	1,563	7,370
TrEMBL	11,442	2,140	8,783
nr	11,453	2,151	8,788
nt	4,754	805	3,768
All	12,049	2,415	9,001

**Table 4:** Summary of simple sequence repeat (SSR) types in the *Tetranychus cinnabarinus* transcriptome.

Repeat motif	Number^a^	Percentage (%)	Repeat motif	Number^a^	Percentage (%)^b^
*Dinucleotide *			*Tetra-nucleotide *		
AC/GT	143		AAAC/GTTT	3	
AG/CT	468		AAAG/CTTT	5	
AT/AT	85		AAAT/ATTT	6	
Total	**696**	30.17%	AAGC/CTTG	2	
*Trinucleotide *			AATC/ATTG	2	
AAC/GTT	310		AATG/ATTC	2	
AAG/CTT	182		ACTC/AGTG	1	
AAT/ATT	182		Total	**21**	0.91%
ACC/GGT	75		*Pentanucleotide *		
ACG/CGT	2		AAAAC/GTTTT	1	
ACT/AGT	8		AAGAG/CTCTT	1	
AGC/CTG	59		AATAT/ATATT	1	
AGG/CCT	48		Total	**3**	0.13%
ATC/ATG	715		*Compound SSR *	4	0.17%
CCG/CGG	2				
Total	**1583**	68.62%			

^a^Number of the specified SSRs detected in unigenes.

^b^The relative percentage of the specified SSRs among the total SSRs.

**Table 5:** Genes involved in insecticide metabolism.

Gene name	Number of sequences with TrEMBL hit
Cytochrome P450	81
Carboxylesterase	28
Glutathione S-transferase	35
Catalase	6
Superoxide dismutase	12
NADH dehydrogenase	28
GABA receptor	9
Cytochrome b	9
Nicotinic acetylcholine receptor	25
Voltage-gated sodium channel	10
Glutamate-gated chloride channel	15
GABA-gated chloride channel	3
Ryanodine receptor	2
Acetylcholinesterase	1
MFS-type transporter	17
ABC transporter	130
Intradiol dioxygenase-like protein	17
Nuclear receptor	15
Major facilitator superfamily	26
Kelch-like protein	33
TWiK family of potassium channels	5
Inward rectifier potassium channel	4
Two pore potassium channel	3
Calcium-activated potassium channel	9
Potassium voltage-gated channel	29
Voltage-dependent calcium channel	11
Calcium release-activated calcium channel	3

**Table 6:** The significantly differentially expressed unigenes between control and *β*-sitosterol-exposed mites.

Number ID	Annotation	Differentially expressed genes		KEGG annotation
		Transcriptome	qPCR	
T2_Unigene_BMK.2524	Carboxyl/cholinesterase	Up	Up	
T2_Unigene_BMK.7122	Carboxypeptidase	Up	Up	—
CL121Contig1	ABC transporter, class C	Up	Up	—
T2_Unigene_BMK.2982	Chloride channel protein 2	Up	Up	K12880
CL5336Contig1	Cytochrome b	Up	Up	K00412
CL1378Contig1	Calphostin	Up	Up	—
T2_Unigene_BMK.8426	Calphostin	Up	Up	—
T1_Unigene_BMK.15610	fatty acid synthase	Down	Down	K00665
CL8369Contig1	Peritrophic membrane chitin binding protein	Up	Up	—
CL8324Contig1	Fibroin	Down	Unchanged	—
T1_Unigene_BMK.9681	Fibroin	Down	Unchanged	—
T3_Unigene_BMK.5391	Fibroin	Down	Unchanged	—
T2_Unigene_BMK.8596	Fibroin	Down	Unchanged	—
CL8591Contig1	Fibroin heavy chain (Precursor)	Down	Down	—
T3_Unigene_BMK.5709	Fibroin heavy chain (Precursor)	Down	Down	—
T1_Unigene_BMK.7441	Putative transcription factor	Up	Up	—
CL9149Contig1	Toll-like receptor 8	Up	Up	—
CL9567Contig1	legumain	Up	Up	—
T1_Unigene_BMK.2690	legumain	Up	Up	K01369
CL2236Contig1	Legumain	Up	Up	K01369
CL710Contig1	beta-mannosidase	Down	Down	K01192
T2_Unigene_BMK.6324	beta-mannosidase	Down	Down	K01192
CL7517Contig1	serine protease	Up	Up	—
T2_Unigene_BMK.3603	serine protease	Up	Up	—
CL1681Contig1	serine proteinase	Up	Up	—
CL7152Contig1	serine protease homologue	Down	Down	—
T2_Unigene_BMK.7468	serine protease homologue	Up	Up	—
T2_Unigene_BMK.3702	serine protease, polymerase	Up	Up	—
T2_Unigene_BMK.6592	serine protease	Up	Up	—
T2_Unigene_BMK.1689	Probable serine/threonine-protein kinase	Up	Up	—
CL2365Contig1	Probable serine/threonine-protein kinase	Up	Up	—
CL5455Contig1	Fibrillar collagen precursor	Up	Up	K06236
T2_Unigene_BMK.12938	Histidine-rich glycoprotein	Down	Down	—
CL10295Contig1	Uncharacterized histidine-rich protein	Down	Down	—
CL3394Contig1	Endonuclease	Down	Down	—
CL7961Contig1	Ribonuclease	Up	Up	—
CL8740Contig1	RNA helicase	Down	Down	—
T2_Unigene_BMK.4212	Ras guanine nucleotide exchange factor	Up	Up	—
T2_Unigene_BMK.17224	helicase	Up	Up	—
CL8128Contig1	deoxyribonuclease	Up	Up	K01158
