# Supplementary material for: Transcriptome Analysis of the Carmine Spider Mite, Tetranychus cinnabarinus (Boisduval, 1867) (Acari: Tetranychidae), and Its Response to β-Sitosterol
Source: Biomed Res Int. 2015 May 11;2015:794718. doi: 10.1155/2015/794718 (PMC4442410; doi:10.1155/2015/794718)
Supplement: Supplementary file 1 — The supplementary materials include seven supplementary tables. In the supplementary tables, the details of primers used for RT-qPCR analyses are provided. Furthermore, the gene numbers of CYP, GSTs and CCEs of Tetranychus cinnabarinus, and comparison of CYP and GSTs among different subgroups are illustrated. In the supplementary tables, gene numbers of differential expressed unigenes of Tetranychus cinnabarinus after exposure to β-sitosterol are also provided. [file 794718.f1.pdf]

Table S1. Primers used for qRT-PCR analyses.

Gene name	Forward primer	Reverse primer
α-tub	ACTACGCTCGTGGCCACTATACAA	ACCAGATCCAGTTCCACCTCCAAA
β-actin	CAGCCATGTATGTTGCCATC	AAATCACGACCAGCCAAATC
T2_Unigene_BMK.2524	ATTGAACACTTGCACTCGGTAT	CCCCTTGAAAATGTAACCTTCGT
CL8369Contig1	CAACTCGTGTATCTTGGTTCTCT	ATGCCCGATGTTTACTTTACC
CL1378Contig1	TAGCTCCTCTTGGTAGTCTTGT	GATAGCTTTACCTCCGACAACA
T2_Unigene_BMK.8426	TAGCTCCTCTTGGTAGTCTTGT	ATAGCTTTACCTCCGACAACA
T1_Unigene_BMK.15610	GTTTCCAAAATCCGAGTCAGT	TTTAGGTTACCAATTTGACCG
CL121Contig1	ATTGAAGCTGAAACTGCCGAAAC	ACTTGAGGCTAAAATGAAACCAC
T2_Unigene_BMK.2982	TTCTCTAGGCAATTGTACTCTC	ACTTTAGACTACGCCAAGCA
CL5336Contig1	GCTCCTCAAAAAGATATTTGTCC	AAACAAATGTGAATTTCAGGG
T2_Unigene_BMK.7122	TGTCTCATTTCAAGCGTGGT	TATTCCTCCGATGTAGCCACT
T1_Unigene_BMK.7441	TGTATTGCGTTATCCGAGCTT	CAGCCAATGAGCGTTCCCAGT
CL9149Contig1	TTAGCAGTGATAGACGACCCAA	ATCCAATCGTGACTTGTGCAT
CL7517Contig1	GGATACTTATTGGCTCGAACCT	TGACCACTCTTTTGCTCGTT
T2_Unigene_BMK.3603	ATACTTATTGGCTCGGACCT	AGATTCTTTATGTTGACCGCTCTTT
CL1681Contig1	TGATCCAGACGATGAAACCTC	ATCGGCATCCAATTCACCAC
CL7152Contig1	CAATGAGCAGCAACAGCGA	GCTTGTTGACTCGGTTCCCT
T2_Unigene_BMK.7468	AAATTATCGGCTCCAGTCCCA	CCTTGCTGGAGTTACTTCTCGT
T2_Unigene_BMK.3702	AGAATTAACTGTTGTCCGTCCC	ACACAATTTACTCCCGTTTGCT
T2_Unigene_BMK.6592	TTTTGTCCGAGAAAGCCTTGT	CTAACCGCTGCTCACTGTGTCACT
CL9567Contig1	AGGCTCCATCAACCTTACCTTC	ATAACACGGATAGGTTGAACAGG
T1_Unigene_BMK.2690	ATATCTACAAATGATGCCGCT	GGGCATCTGAGCAATAAC
CL2236Contig1	AAACATTGGCTTGATGACGA	ACTCCTTTGGCTGATTCGTTG
CL5455Contig1	TCCTTTCAAACCAGGCATTCCA	CTAGTGGAAAACCCGGTCCT
T2_Unigene_BMK.1689	TCATTTCGAGATTTGGCGTTCCTT	ATATTCTCAAAGGTGGACGCAA

CL2365Contig1	ACTCCAGCTTCATACGTCTCC	CGCTGATCTTCTTTGATGCTGTCT
CL8324Contig1	TGGACACATCAGGCATT	AAAGCCCTGGATAAACAC
T1_Unigene_BMK.9681	ATGCTGCAGGGATAACTACAAC	CACACTTTATACGTCGCTGTC
T3_Unigene_BMK.5391	GTCTTCATTAACTTGGCTTGTCT	TTCTTCACAGGCAACAAGCTC
T2_Unigene_BMK.8596	TTCTTCACAGGCAACAAGCTC	TCATTAACTTGGCTTGTCTGG
CL8591Contig1	TAACCTTGCTGCTACCGGA	GAAAGCACCTAAACCAAGTCCA
T3_Unigene_BMK.5709	ACCCATTTAAAATAGCCTCGCA	ATGAACCAGCAGTTTTGATTCC
T2_Unigene_BMK.12938	CCGTGATGATCCTTATGCTTG	GATCTTTGCTCTTCTCGCCTT
CL10295Contig1	GATTTCCAAAACGTCCGGCAG	CCAAACAGAGGATTCGGCAAC
CL3394Contig1	TCGACTCGCAAACGCCACT	ATTACTAACCCAACTACGCTGTGA
CL7961Contig1	AATGGAAGAAACACGGAAC	GGTTTGATTCCAGCAGTT
CL8740Contig1	TTTCGCAAGCATTTTCGTCCC	GAGCCAATTCATTCAATCGTCCA
T2_Unigene_BMK.4212	AATGTTTGATGAGCTGACTTTG	GTTCGGAATTCTGTCAACAACC
T2_Unigene_BMK.17224	AACCCTCCAGCTCAACAATC	GGTTTGCTTTCATCTTTGGCT
CL8128Contig1	CAAATTACCTCGAGAAAGCCAC	TTGACCCCACATTGAGTCC
CL710Contig1	AAAGACGACAGTGATTATGCC	TGAAGTCAAGCCAAACAAATAG
T2_Unigene_BMK.6324	ACAATCCCTACGCTTATGCTC	CCCATCCCCACATCATCTTTC

Table S2. Comparison of CYP gene number in Insecta, Crustacea and *T. urticae*.

	CYP2 clan	mitochondrial CYP clan	CYP3 clan	CYP4 clan	Total
**Insecta**					
Drosophila melanogaster	7	11	36	32	88
Anopheles gambiae	10	9	40	46	105
Aedes aegypti	12	9	82	57	160
Bombyx mori	7	12	30	36	85
Apis mellifera	8	6	28	4	46
Nasonia vitripennis	7	7	48	30	92
Tribolium castaneum	8	9	72	45	134
Acyrthosiphon pisum	10	8	23	23	64
Pediculus humanus	8	8	11	9	36
**Crustacea**					
Daphnia pulex	20	6	12	37	75
**Acari**					
Tetranychus urticae	48	5	10	23	86
T. cinnabarinus	49	3	7	22	81

Data are derived from Grbic *et al.* and this study.

Table S3. Gene numbers of CYP sequences of *T. cinnabarinus*.

gene ID	gene ID	gene ID	gene ID	gene ID
CL6302Contig1	CL7962Contig1	T2_Unigene_BMK.15594	T2_Unigene_BMK.2540	T1_Unigene_BMK.12473
CL7880Contig1	T2_Unigene_BMK.5372	T1_Unigene_BMK.16633	CL8013Contig1	T2_Unigene_BMK.3737
CL10156Contig1	T2_Unigene_BMK.14385	CL7701Contig1	T3_Unigene_BMK.3421	CL5611Contig1
CL1143Contig1	T2_Unigene_BMK.8419	T2_Unigene_BMK.16031	T2_Unigene_BMK.11945	CL5259Contig1
CL2950Contig1	T2_Unigene_BMK.14948	CL1520Contig1	T3_Unigene_BMK.5800	T2_Unigene_BMK.6710
T3_Unigene_BMK.5585	T2_Unigene_BMK.11299	CL533Contig2	T2_Unigene_BMK.8805	CL7566Contig1
T2_Unigene_BMK.16778	T3_Unigene_BMK.9327	CL891Contig1	CL4296Contig1	T3_Unigene_BMK.1196
CL299Contig1	T2_Unigene_BMK.14798	T1_Unigene_BMK.15369	T2_Unigene_BMK.14016	T1_Unigene_BMK.3580
T2_Unigene_BMK.8914	T1_Unigene_BMK.9503	CL7269Contig1	CL2243Contig1	CL2387Contig1
CL5888Contig1	T2_Unigene_BMK.5358	T3_Unigene_BMK.2483	CL8423Contig1	T1_Unigene_BMK.8630
T2_Unigene_BMK.7768	CL539Contig1	T1_Unigene_BMK.12554	CL6061Contig1	T1_Unigene_BMK.9148
T1_Unigene_BMK.5609	CL3603Contig1	CL3793Contig1	CL9736Contig1	T2_Unigene_BMK.2805
CL934Contig1	T1_Unigene_BMK.2070	CL8789Contig1	CL8083Contig1	CL1685Contig1
CL8897Contig1	CL9195Contig1	T3_Unigene_BMK.1479	T2_Unigene_BMK.5369	CL8107Contig1
CL3234Contig1	T2_Unigene_BMK.3919	CL8768Contig1	T2_Unigene_BMK.13135	T2_Unigene_BMK.12265
T1_Unigene_BMK.8438	T3_Unigene_BMK.15246	CL9813Contig1	CL2486Contig1	CL5098Contig1
T2_Unigene_BMK.7326				

Table S4. An overview of GSTs and the number of genes belonging to different subgroups.

GSTfamily	*D. melanogaster*	*A.Gambiae*	*A.mellifera*	*N. vitripennis*	*T. castaneum*	*B. mori*	*H. sapiens*	*T.urticae*	*T. cinnabarinus*
alpha	-	-	-	-	-	-	5	-	-
delta	11	12	1	5	3	4	-	16	9
epsilon	14	8	-	-	19	8	-	-	-
mu	-	-	-	-	-	-	5	12	10
pi	-	-	-	-	-	-	1	-	-
omega	5	1	1	2	4	4	2	2	3
sigma	1	1	4	8	7	2	1	-	-
theta	4	2	1	3	1	1	2	1	3
zeta	2	1	1	1	1	2	1	-	2
unknown	-	3	-	-	-	2	-	-	7
kappa	-	-	-	-	-	-	-	1	1
Total	37	28	8	19	35	23	17	31	35

Data are derived from Grbic *et al.* and this study.

Table S5. Gene numbers of GST sequences of *T. cinnabarinus*.

gene ID	gene ID	gene ID	gene ID	gene ID
T2_Unigene_BMK.3207	T2_Unigene_BMK.8773	CL7870Contig1	CL677Contig1	T1_Unigene_BMK.3507
CL1968Contig1	CL7748Contig1	CL2232Contig1	CL5326Contig1	CL8336Contig1
T2_Unigene_BMK.1887	T1_Unigene_BMK.3657	CL1977Contig1	T3_Unigene_BMK.16184	T1_Unigene_BMK.6540
CL755Contig1	T1_Unigene_BMK.3259	T1_Unigene_BMK.3656	CL7599Contig1	T3_Unigene_BMK.3671
T2_Unigene_BMK.4442	CL8422Contig1	T1_Unigene_BMK.17845	CL5410Contig1	CL2068Contig1
CL4394Contig1	T3_Unigene_BMK.3480	T2_Unigene_BMK.1888	CL4707Contig1	T1_Unigene_BMK.4118
T2_Unigene_BMK.17617	T1_Unigene_BMK.7739	T1_Unigene_BMK.4706	T2_Unigene_BMK.3986	T2_Unigene_BMK.2893

Table S6. Gene numbers of CCE sequences of *T. cinnabarinus*.

gene ID	gene ID	gene ID	gene ID
T2_Unigene_BMK.3207	CL8507Contig1	T1_Unigene_BMK.8116	CL4302Contig1
CL4095Contig1	T2_Unigene_BMK.8535	CL1454Contig1	CL8507Contig1
T1_Unigene_BMK.4410	CL9410Contig1	T3_Unigene_BMK.2647	CL7512Contig1
T3_Unigene_BMK.4715	CL9457Contig1	CL3357Contig1	T3_Unigene_BMK.10832
T3_Unigene_BMK.5460	T3_Unigene_BMK.10549	CL3647Contig1	T3_Unigene_BMK.5460
CL8135Contig1	T3_Unigene_BMK.13402	CL8507Contig1	T1_Unigene_BMK.17676
T2_Unigene_BMK.8420	T3_Unigene_BMK.13492	T2_Unigene_BMK.2524	T2_Unigene_BMK.5720

Table S7. Gene numbers of differential expressed unigene sequences of *T. cinnabarinus* after exposure to β-Sitosterol.

gene ID	gene ID	gene ID	gene ID	gene ID
CL10201Contig1	CL5455Contig1	CL9534Contig1	T2_Unigene_BMK.12938	T2_Unigene_BMK.6592
CL10295Contig1	CL5462Contig1	CL9567Contig1	T2_Unigene_BMK.13937	T2_Unigene_BMK.7122
CL121Contig1	CL5765Contig1	T1_Unigene_BMK.14313	T2_Unigene_BMK.14070	T2_Unigene_BMK.7273
CL124Contig2	CL710Contig1	T1_Unigene_BMK.15610	T2_Unigene_BMK.15551	T2_Unigene_BMK.7468
CL1378Contig1	CL7152Contig1	T1_Unigene_BMK.1995	T2_Unigene_BMK.16222	T2_Unigene_BMK.8426
CL1478Contig1	CL7387Contig1	T1_Unigene_BMK.2690	T2_Unigene_BMK.1689	T2_Unigene_BMK.8596
CL1681Contig1	CL7517Contig1	T1_Unigene_BMK.2837	T2_Unigene_BMK.17115	T2_Unigene_BMK.8948
CL187Contig2	CL7961Contig1	T1_Unigene_BMK.3279	T2_Unigene_BMK.17224	T2_Unigene_BMK.9070
CL2236Contig1	CL8023Contig1	T1_Unigene_BMK.5027	T2_Unigene_BMK.17291	T3_Unigene_BMK.2860
CL2285Contig1	CL8128Contig1	T1_Unigene_BMK.5829	T2_Unigene_BMK.2099	T3_Unigene_BMK.5391
CL2338Contig1	CL8324Contig1	T1_Unigene_BMK.7441	T2_Unigene_BMK.2468	T3_Unigene_BMK.5709
CL2365Contig1	CL8332Contig1	T1_Unigene_BMK.9681	T2_Unigene_BMK.2629	T3_Unigene_BMK.5786
CL2399Contig1	CL8369Contig1	T1_Unigene_BMK.9980	T2_Unigene_BMK.2982	T3_Unigene_BMK.6006
CL2518Contig1	CL8591Contig1	T2_Unigene_BMK.11334	T2_Unigene_BMK.3603	T3_Unigene_BMK.8083
CL3120Contig1	CL8619Contig1	T2_Unigene_BMK.11446	T2_Unigene_BMK.3702	T3_Unigene_BMK.946
CL3394Contig1	CL8740Contig1	T2_Unigene_BMK.11922	T2_Unigene_BMK.4212	
CL502Contig1	CL9149Contig1	T2_Unigene_BMK.11961	T2_Unigene_BMK.476	
CL5296Contig1	CL9211Contig1	T2_Unigene_BMK.12082	T2_Unigene_BMK.6163	
CL5336Contig1	CL9273Contig1	T2_Unigene_BMK.12583	T2_Unigene_BMK.6324	
